# Dolenc approach for clipping of giant C6 and C7 segment aneurysms of the internal carotid artery

**DOI:** 10.3389/fsurg.2023.1222386

**Published:** 2023-08-21

**Authors:** Hongwei Zhang, Wei Liu, Yan Gu, Aimin Li, Dong Zhang

**Affiliations:** ^1^ Department of Neurosurgery, The Affiliated Lianyungang Hospital of Xuzhou Medical University, Lianyungang, China; ^2^ Department of Neurosurgery, Beijing Tiantan Hospital Affiliated to Capital Medical University, Beijing, China; ^3^ Department of Neurosurgery, Beijing Hospital, National Center of Gerontology, Institute of Geriatric Medicine, Chinese Academy of Medical Sciences, Beijing, China

**Keywords:** Dolenc approach, giant aneurysm, internal carotid artery, ophthalmic aneurysm, anterior clinoid process, posterior communicating artery aneurysm

## Abstract

**Objective:**

Surgical treatment for giant aneurysms of the ICA-ophthalmic segment (C6) and communicating artery segment (C7) is a challenge for neurosurgeons because of their high risks and poor outcomes. We aim to explore the advantages and disadvantages of the Dolenc approach in the treatment of giant C6–C7 segment aneurysms.

**Methods:**

We retrospectively reviewed the clinical data of 13 cases with giant C6 aneurysms and 4 cases with giant C7 aneurysms treated with the Dolenc approach.

**Results:**

All 17 cases of aneurysms were clipped successfully using the Dolenc approach, of which, 1 case with ipsilateral MCA occlusion underwent extracranial-intracranial artery bypass after the aneurysm clipping. Regarding clinical outcomes, six out of nine cases with preoperative visual impairment improved after surgery, two cases saw no change, and one case deteriorated. Of all the cases, one had new-onset vision loss, four had new-onset oculomotor paralysis, three had surgical side cerebral infarction, and two had diabetes insipidus. DSA or CTA examination within 2 weeks after surgery showed that all aneurysms were completely clipped without residual. After a follow-up of 9–12 months, 17 patients were evaluated based on GOS and CTA examination. A total of 14 cases had GOS 5 scores, 2 cases had GOS 4 scores, 1 case had GOS 2 scores, and no cases had death. None of the patients had recurrence based on CTA examinations.

**Conclusion:**

Through the Dolenc approach, we could achieve more operation space and expose clinoid segments for temporary occlusion. Therefore, the Dolenc approach was shown to be a safe, effective, and feasible treatment for giant C6–C7 aneurysms.

## 1. Introduction

The giant intracranial aneurysm is a special type of intracranial aneurysm with a diameter larger than 2.5 cm. They constitute 3%–5% of all intracranial aneurysms, and the ophthalmic segment (C6) and communicating artery segment (C7) of the internal carotid artery (ICA) are common locations for giant intracranial aneurysms (1–5). Due to the large size, high tension, wide neck, thrombus, calcification, complex surrounding structures, and extended parent artery of giant C6–C7 aneurysms, both clipping and interventional treatments are associated with increased difficulties and risks.

In recent years, more and more intracranial aneurysms have been treated with interventional therapy. However, it was still a challenge for endovascular therapy to occlude the giant aneurysm and preserve the patency of the parent artery (6–9). Although improvement of the interventional technique and application of new materials compensate for the aforementioned defects to some extent (10–12), intervention still has some unsatisfactory aspects, such as complete occlusion rate, high residual rate, and postoperative risk of infarction, removal of the occupying effect and improvement of visual impairment, and long-term use of anticoagulants (13–17). Therefore, clipping is still an effective and safe way to treat giant aneurysms.

In 1985, Professor Vinko Dolenc proposed a method, namely, extradural drilling of bony structures, such as the ACP, combined with subdural approach, which has a satisfactory effect in treating ophthalmic aneurysms (18, 19). Through this approach, we can get a better surgical field, expose the ophthalmic and clinoid segments of the ICA, expose and protect the surrounding structures, reduce temporal lobe injury, and improve the safety of the surgery. This approach is also suitable for the surgical treatment of giant C6–C7 aneurysms. The purpose of this study was to review our recent experiences using the Dolenc approach to surgically clip giant C6–C7 aneurysms. We aimed to explore the techniques involved in the Dolenc approach, assess the effectiveness of the Dolenc approach in the surgical clipping of giant C6–C7 aneurysms, and evaluate the outcomes of patients with giant C6–C7 aneurysms who underwent this treatment.

## 2. Materials and methods

### 2.1. General materials

From January 1, 2015 to December 31, 2018, a total of 17 cases with giant C6–C7 aneurysms treated by the Dolenc approach were retrospectively reviewed. Among the 17 cases, 13 had giant C6 aneurysms, including one male and 12 females with an average age of 51.2 years (range: 33–70 years). Five cases had ruptured aneurysms and eight had unruptured aneurysms. Three cases had recurrent aneurysms after endovascular coiling. Eight patients had vision impairment preoperatively, of which one case was bilateral and seven cases were ipsilateral. Six patients had hypertension, two had coronary disease, and two had thyroid diseases.

Four cases of giant C7 aneurysms were enrolled, of which, one was male and three were female, with an average age of 62.3 years old (range: 58–71 years old). One patient had a ruptured aneurysm, and the remaining three patients had unruptured aneurysms. One case had a recurrent aneurysm after interventional treatment and the other case was recurrent after clipping and interventional treatment. One patient had preoperative oculomotor paralysis and another patient had hypertension (HBP) and coronary disease (CAD). Additionally, there was a patient with a history of cerebral haemorrhage, another with diabetes mellitus, and a third with a dural arteriovenous fistula.

All 17 cases were diagnosed by CTA or DSA, with aneurysm diameters larger than 2.5 cm. Giant C6 aneurysms were grouped by size: seven cases were 2.5–3.0 cm, three were 3.0–3.5 cm, two were 3.5–4.0 cm, and one was >4.0 cm. Three cases were recurrent after interventional treatment, and one combined ipsilateral MCA occlusion. Giant C7 aneurysms were grouped: one was 2.5–3.0 cm, two were 3.0–3.5 cm, and one was >4 cm. One of them was a recurrent aneurysm after intervention, and the other was a recurrent aneurysm after intervention and surgery. A summary of patient characteristic information is shown in Table 1. All patients signed an institutional consent form to undergo surgery and agreed to publish the results of the surgery for use in various types of medical publications.

**Table 1:** Clinical characteristics of 13 cases of giant ophthalmic artery aneurysms and 4 cases of giant posterior communicating aneurysms.

	Ophthalmic artery aneurysms	Posterior communicating aneurysms
Number of patients	13	4
Age (years)		
Range	33–70	58–71
Mean	51.2	62.3
Sex		
Male	1	1
Female	12	3
Side		
Left	7	1
Right	6	3
Aneurysm size		
2.5–3.0 cm	7	1
>3.0 cm, ≤3.5 cm	3	2
>3.5 cm, ≤4.0 cm	2	0
>4.0 cm	1	1
Hunt and Hess grade		
0	8	2
1–3	4	2
4–5	1	0
Visual disturbance	9	0
Treatment before		
Coiling	3	1
Clipping	0	0
Coiling and clipping	0	1

### 2.2. Surgical methods

**Figure 1:**
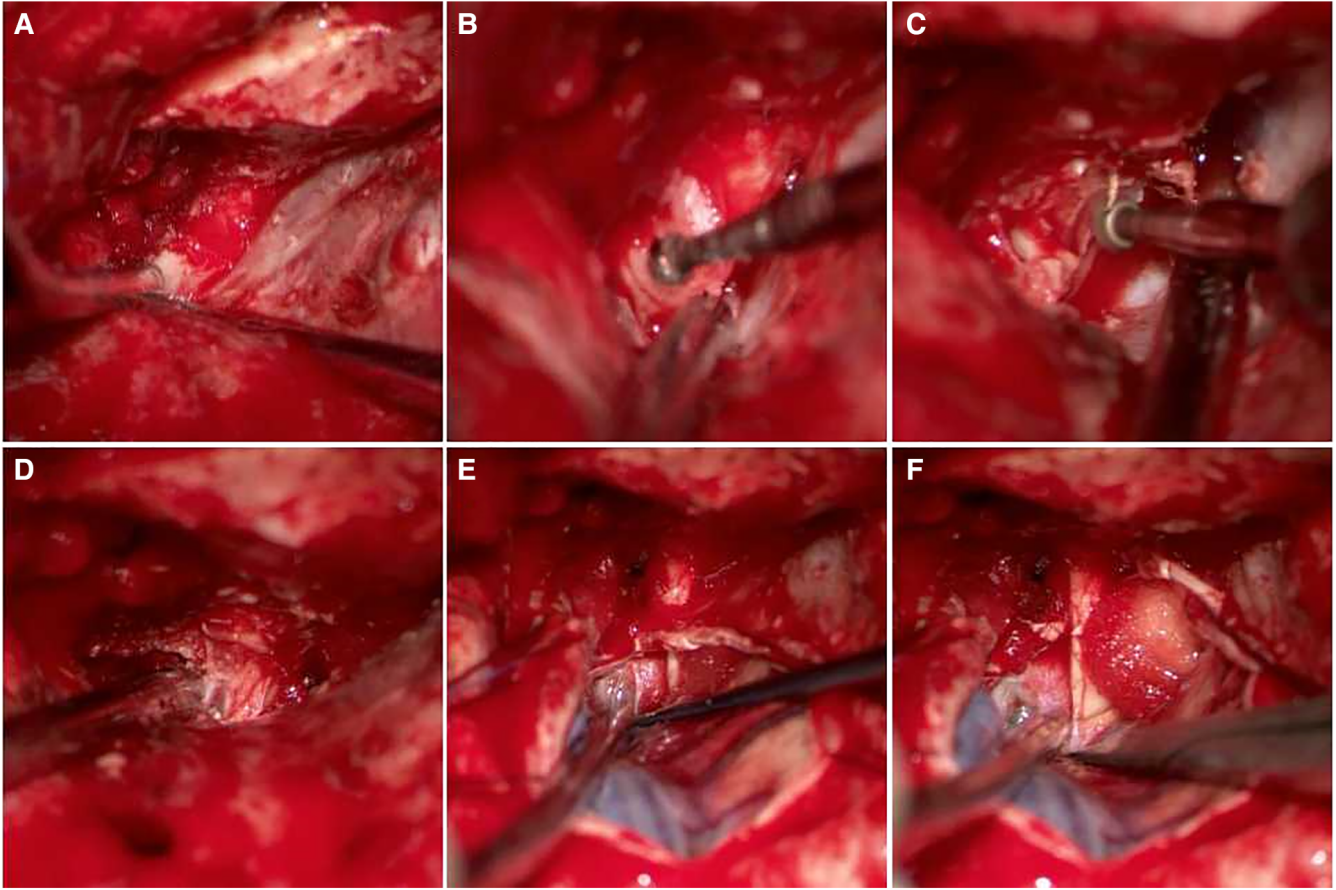
The process of drilling ACP and opening dura mater. (**A**) Removed sphenoid ridge, Exposed the ACP; (**B**) drilled anterolateral wall of the ACP; (**C**) drilled superolateral wall of the optic canal; (**D**) removed the ACP and superior wall of the optic nerve and cavernous sinus was visible; (**E**) sheared dura mater along lateral fissure; (**F**) opened optic nerve dura sheath and aneurysm is visible.

All aneurysms were clipped by a single senior professor using the Dolenc. The steps were as follows and as shown in Figure 1. Patients receiving general anaesthesia were placed in the supine position with their heads turning to the contralateral side 20–30 degrees, angling back 10–15 degrees, and fixing by the head frame. Bone flap and Partial sphenoid ridge were removed by standard pterional craniotomy and dura mater was opened at the frontotemporal lobe on both sides of the ACP. We then proceeded to drill the anterolateral wall of the ACP and superior lateral wall of the optic canal with a small high-speed burr until an “eggshell” appearance was left. Subsequently, we separated and removed the “eggshell” bone from the inferoanterior portion of the optic strut with a combination of small high-speed burr and micro rongeur. We then opened the dura mater along the lateral fissure and split the arachnoid pool to release cerebrospinal fluid. Finally, we opened the optic nerve dura sheath and distal ring to expose the C7 segment, C6 segment (or clinoid segment) of ICA, and aneurysmal neck for clipping. Temporary occlusion of the clinoid segment could be performed when necessary.

## 3. Results

All 17 cases with giant aneurysms were successfully clipped, 3 of which were ruptured during surgery and were clipped after temporary occlusion, and 1 had ipsilateral MCA occlusion, requiring extracranial-intracranial artery bypass thereafter. Of the 17 cases, 5 had recurrent aneurysms after interventional therapy, and 4 of them had embolization resection. Of the nine cases with preoperative visual impairment, six cases had visual improvement, two cases of ophthalmoplegia did not improve. One patient experienced newly developed vision loss and recovered after treatment. Of the four oculomotor paralysis patients, three improved after conservative treatment. Meanwhile, three patients had surgical side cerebral infarction with decreased muscle strength in the contralateral limbs, and two patients had diabetes insipidus and electrolyte imbalance, from which they had completely recovered upon discharge from hospital. DSA or CTA examination was conducted 2 weeks after surgery. All of the 17 aneurysms were completely clipped without any residual. After a follow-up of 9–12 months, 17 patients were evaluated by GOS and CTA examination, in which 14 patients had a GOS score of 5, 2 had a GOS score of 4, 1 had a GOS score of 2, and no patients had death. None of the patients had recurrence based on CTA examinations. No patient in our study showed intracranial infection or cerebrospinal fluid leakage (Table 2).

**Table 2:** Clinical outcomes of 13 cases of giant ophthalmic artery aneurysms and 4 cases of giant posterior communicating aneurysms.

	Ophthalmic artery aneurysms	Posterior communicating aneurysms
Visual acuity		
Improved	7	1
Unchanged	2	0
Worsened	1	0
Oculomotor nerve injury	3	1
Diabetes insipidus	2	0
Cerebral infarction	1	2
CSF leakage	0	0
Rehaemorrhagia	0	0
Intracranial infection	0	0
Glasgow Outcome Scale		
5	11	3
4	2	1
3	0	0
2	1	0
1	0	0

### 3.1. Illustrated cases

**Figure 2:**
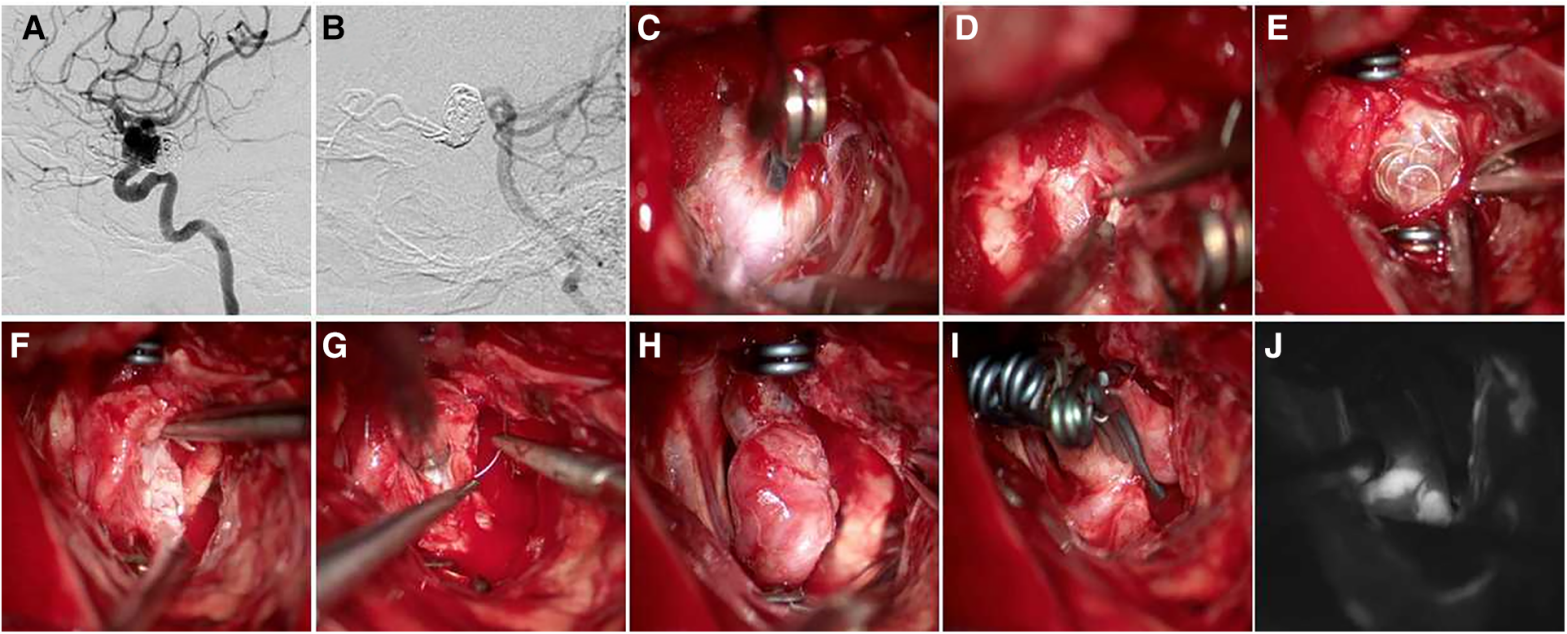
A 61-year-old female patient was diagnosed with a recurrent aneurysm, with a history of clipping surgery and interventional treatment. (**A,B**) Pre-operative DSA shows recurrent C7 aneurysm after clipping and interventional treatment; (**C**) the aneurysm and clip were visible during surgery; (**D**) exposed clinoid segment of the ICA; (**E**) after temporary occlusion of the clinoid segment of the ICA and the distal portion of the parent artery, embolism was visible; (**F**) removal of embolic material; (**G,H**) sutured aneurysm sac; (**I**) clipped aneurysm with three clips side by side; (**J**) intraoperative ICG showed absence of aneurysm, distal parent artery was patent.

Case 1 Female, 61 years old, with a medical history of posterior communicating aneurysm who underwent clipping surgery 7 years prior and interventional treatment 3 years ago. The check-up revealed a recurrent aneurysm. Details of the surgical procedure can be seen in Figure 2.

**Figure 3:**
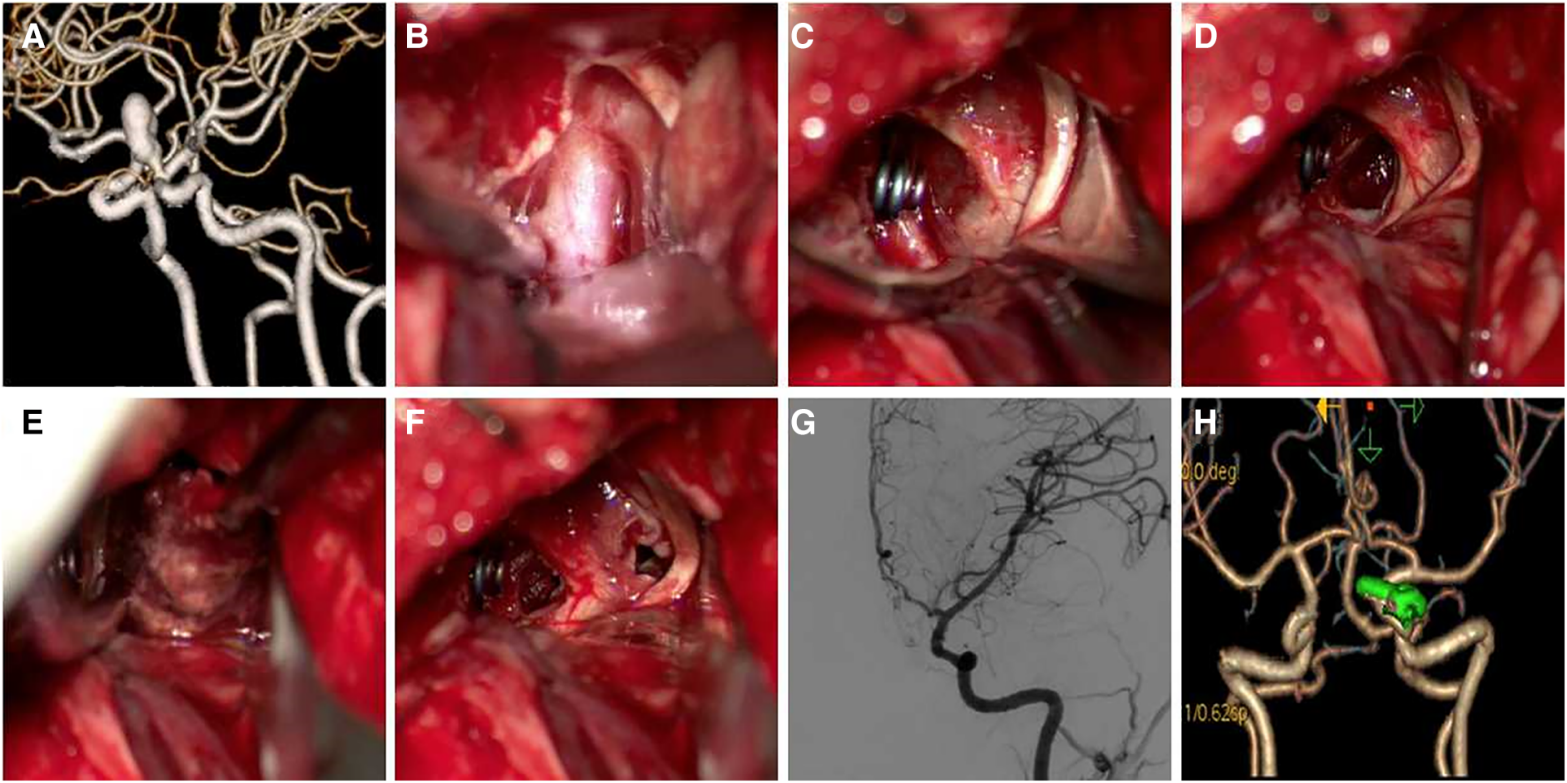
A 33-year-old female patient presented with progressive left-eye vision loss. (**A**) CTA shows C6 aneurysm with narrow neck and long sac; (**B**) exposed aneurysm neck; (**C**) clipped aneurysm neck, optic nerve is obviously compressed; (**D–F**) removed thrombus between space I and II and partially removed aneurysm sac for decompression; (**G,H**) postoperative DSA and CTA follow-up (Patient's vision improved after surgery. Patient had post-operative diabetes insipidus that improved after treatment).

Case 2 Female, 33 years old, left eye vision progressively decreased 8 months ago, which was diagnosed with C6 aneurysm. Details of the surgical procedure can be seen in Figure 3. Patient's vision improved after surgery. Patient had post-operative diabetes insipidus that improved after treatment.

**Figure 4:**
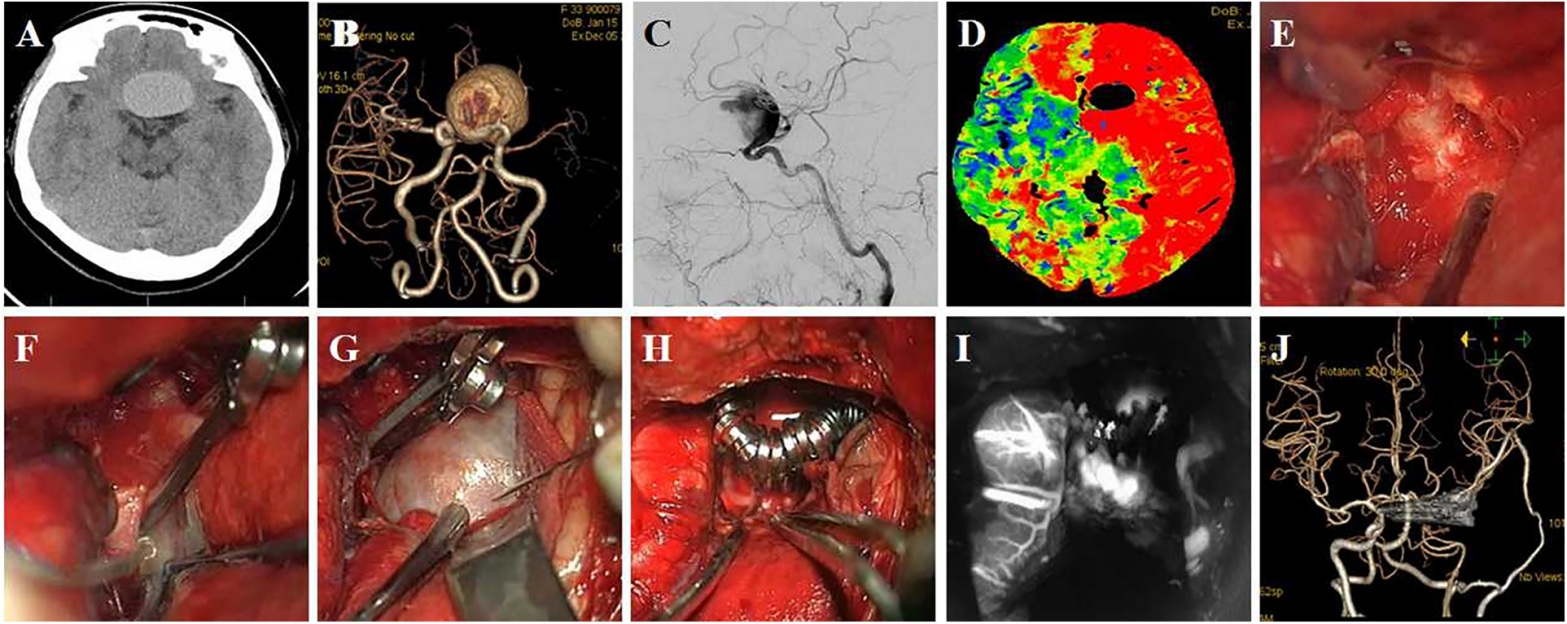
A 33-year-old female patient presented with recurrent headache, which had lasted 1 year. (**A**) Preoperative CT shows a sellar space-occupying lesion; (**B,C**) CTA and DSA show a giant C6 aneurysm and non-visualization of ipsilateral MCA; (**D**) CTP shows hypo-perfusion on ipsilateral ICA distribution area; (**E**) exposed clinoid segment of ICA and the distal ring was seen; (**F**) difficulty in clipping aneurysm neck due to calcification; (**G**) aneurysm puncture after temporary occlusion; (**H**) the aneurysm was clipped by multiple clips; (**I**) ICG in surgery shows disappearance of aneurysm and patency of parent artery; (**J**) postoperative CTA shows absence of the aneurysm, and superficial temporal artery-middle cerebral artery anastomosis is patent.

Case 3 Female, 33 years old complained of recurrent headache for 1 year and revealed a giant C6 aneurysm and non-visualization of ipsilateral MCA. She was treated by aneurysm clipping and superficial temporal artery-middle cerebral artery anastomosis. The surgical procedure is shown in Figure 4.

**Figure 5:**
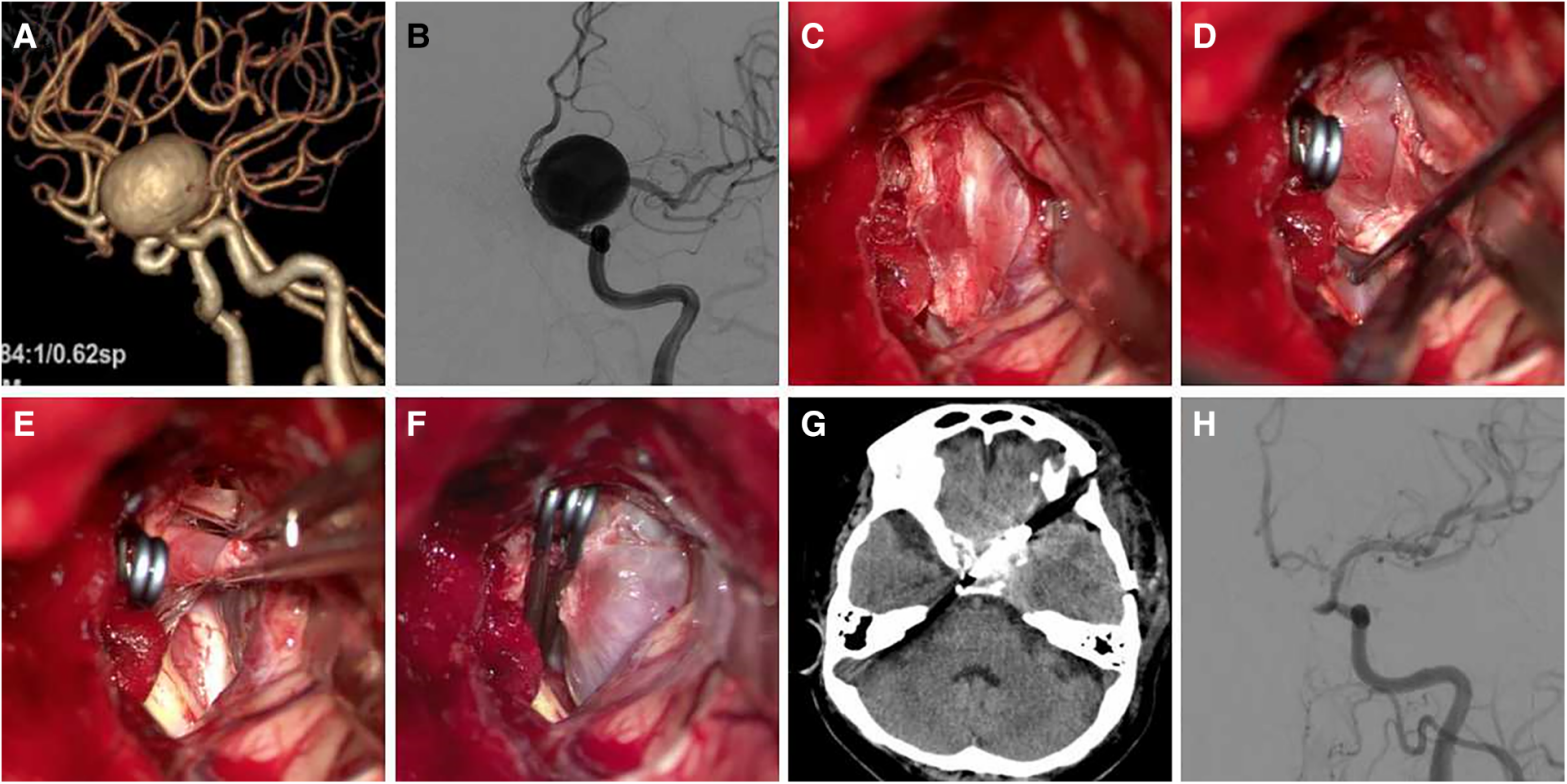
A 60-year-old female patient was diagnosed with giant C6 aneurysm. (**A,B**) Pre-operative CTA and DSA show a C6 aneurysm, the body point to dorsal-medial direction; (**C**) exposure aneurysm and clinoid segment of the ICA; (**D**) temporary occlusion of the clinoid segment of the ICA; (**E,F**) aneurysm neck is not wide, the aneurysm neck was completely clipped using two clips; (**G,H**) postoperative CT and DSA follow-up.

Case 4 Female, 60 years old, was diagnosed with a giant C6 aneurysm in a physical examination 2 months previously. The patient had no pre-operative symptoms. The aneurysm was punctured to relieve pressure but was not removed (Figure 5).

**Figure 6:**
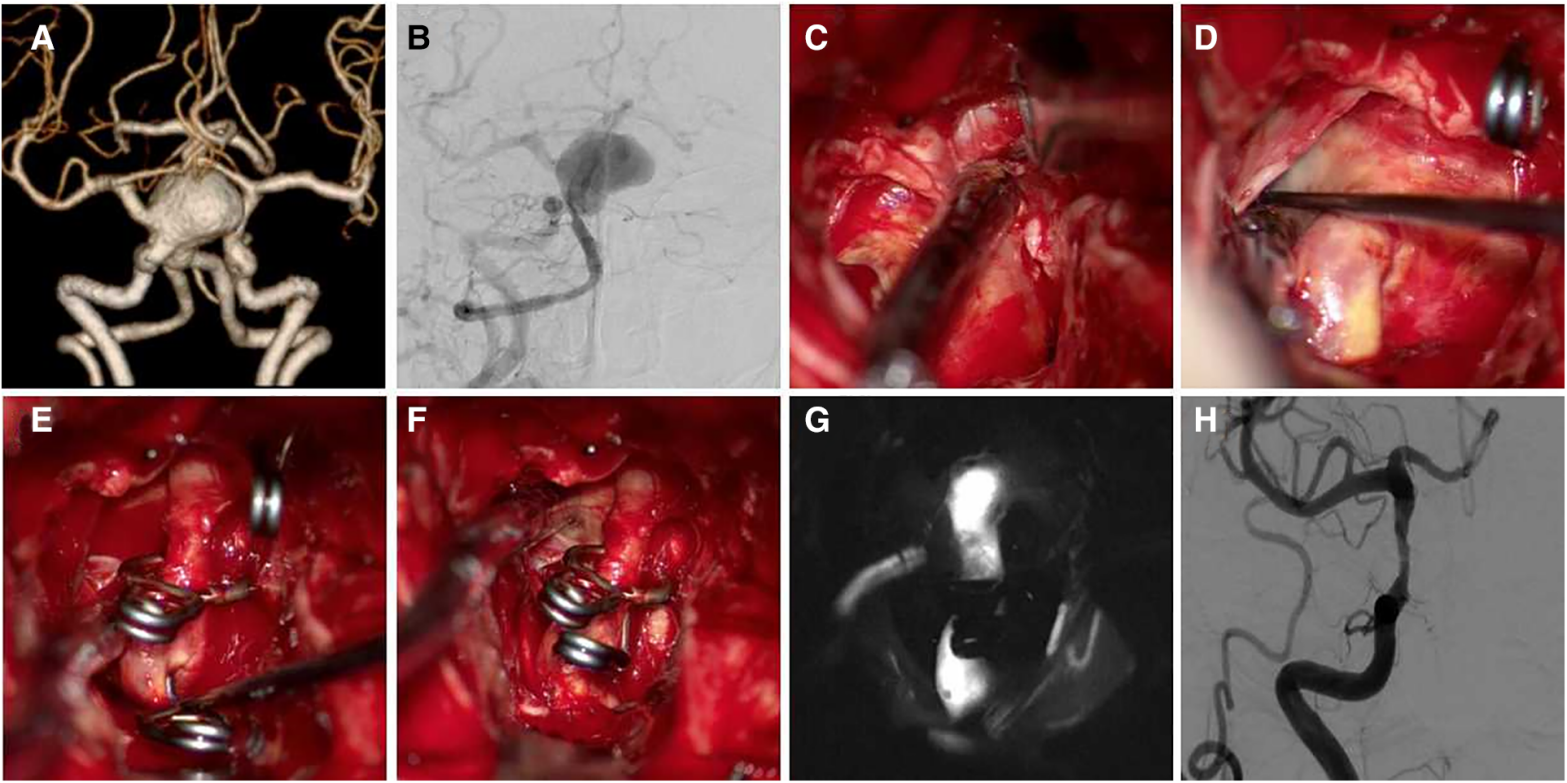
A 61-year-old patient presented with blurred vision in the right eye, which had lasted 6 months. (**A,B**) Preoperative CTA or DSA showed an ophthalmic aneurysm located on the ventral-medial wall of the parent artery; (**C**) exposure and temporary occlusion of the clinoid segment of the ICA; (**D**) the parent artery is dilated, and it is difficult to distinguish the neck of the aneurysm; (**E**) “T” shaped clip was used to clip the aneurysm, and the aneurysm residual at the distal end was seen; (**F**) A fenestrated-angled clip was used to clip the residual; (**G,H**) ICG in surgery, DSA follow-up showed patency and mild stenosis of parent artery.

Case 5 Female, 61 years old, presented with blurred vision in the right eye 6 months previously and an ophthalmic aneurysm was visible. The patient was treated by aneurysm clipping. DSA follow-up showed patency and mild stenosis of the parent artery (Figure 6).

## 4. Discussion

Giant aneurysms of the ICA are a special type of intracranial aneurysm with a high annual rupture rate. According to the literature, the rupture rate of an intracranial aneurysm is closely related to its diameter. Annual rupture rates for 3–4, 5–6, 7–9, 10–24, and ≥25 mm are 0.14%, 0, 1.19%, 1.07%, and 10.61%, respectively (20). Indeed, giant aneurysms' annual rupture rates are much higher than other aneurysms. The mortality rate within 1 year after conservative treatment of giant aneurysm rupture is extremely high and is much higher than surgical treatment and interventional treatment. One study followed eight patients with conservatively managed giant ruptured aneurysms, and they had a 100% mortality rate (21). Therefore, giant aneurysms of the ICA require surgical treatment in the early stage, regardless of whether they are ruptured or not.

The giant C6–C7 segment of the ICA is a common location for giant aneurysms. Due to the size, location, and complicated surrounding structures, aneurysm clipping in this area is a challenge for neurosurgeons. Aneurysm clipping difficulties and risks are due to the following: (1) Aneurysm sizes are substantial and the aneurysm is obstructed by the ACP. Thereby, it is necessary to remove the ACP for more exposure. (2) In addition, there is high risk and difficulty associated with the treatment of an aneurysm after intraoperative rupture, especially for aneurysms on the dorsal wall of the parent artery during extradural surgery. It is necessary to expose the cervical carotid artery or balloon-assisted occlusion of the proximal ICA before craniotomy. (3) The aneurysm often compresses the ophthalmic nerve and cavernous sinus and changes the parent artery's route, which results in limited space for surgery, making it difficult to expose the aneurysm neck. (4) Most giant aneurysm necks are wide, with vasodilation of the parent artery, which means that the aneurysm often fuses with parent arteries, and it may sometimes be challenging to distinguish them even under the microscope. Therefore, it is difficult to use a single clipping. In most cases, combined clipping or clipping after reshaping is necessary. (5) Giant aneurysms have a close relationship with the posterior communicating artery, ophthalmic artery, and superior hypophyseal artery. Therefore, these arteries are susceptible to iatrogenic injury during surgery. (6) Giant aneurysms often have calcification and thrombosis. (7) For those aneurysms that have mass effect, thrombus or previous coil materials within the aneurysm should be removed to reduce compression. (8) For recurrent cases after clipping, it is more difficult to expose or clip the aneurysm due to “messy” anatomical structures.

The Dolenc approach has several advantages in the treatment of giant C6–C7 segment aneurysms of the ICA. Firstly, removal of ACP via the extradural approach is safer and has a lower risk of temporal lobe injury. Secondly, it allows for the removal of bony structure, with better exposure of lesions, better view during surgery, and increased surgical space. Thirdly, it allows for better protection of important blood vessels and nerves in the surgical area and exposes more of the proximal portion of the ICA, making it easier to clip the aneurysm and achieve temporary occlusion of the parent artery. Indeed, according to the literature, drilling of the ACP can increase the exposure of the internal section of the ICA by 110% and the external section of the C6 segment by 60% (22).

Treatment of ACP is a critical step of the Dolenc approach. Several tips to perform the procedure are as follows. (1) Perfect preoperative imaging examination to see if there is ACP variation since variation can increase the level of intraoperative difficulty and risk. (2) When separating the lateral dura mater from the ACP, there is a lateral frontotemporal dural fold, which can be sharply cut and should not exceed 5 mm to prevent injury of the cavernous sinus nerve. (3) For removal of the ACP, the first step is to use a small high-speed drill, resulting in an “egg-shell” appearance of the ACP bones, followed by the use of the micro-rongeur to remove the bone to prevent mechanical damage. (4) Continuous flush when drilling the lateral wall of the optical canal and anterolateral wall of the ACP to avoid heat injury of the optical nerve and oculomotor nerve. (5) If separated ACP adheres to the dura mater, it should be removed by blunt dissection, and forcible pulling should be avoided. (6) When removing the ACP, if the air cell is open, try to keep the mucosal integrity and seal with bone wax. If the mucosa ruptures, suture it and seal it with bone wax to prevent postoperative cerebrospinal fluid leakage. (7) Avoid excessive stress on the dura mater when performing separation and drilling of the ACP, since it could result in aneurysm rupture when it is close to the ACP or dura, or when the pressure is transferred to the aneurysm.

Clipping giant C6–C7 segment aneurysms using the Dolenc approach still has high associated risk. Therefore, it is important to perform an adequate preoperative evaluation. The patient's general condition should be evaluated, and imaging examinations (CT, CTA, CTP, MR, DSA) should be performed to determine the size, morphology, location, projection, and neck of the aneurysm. It is also necessary to know the anatomical relationship of the aneurysm with the parent artery, as well as its branches, ACP and cavernous sinus, thrombus or calcification of the aneurysm, cerebral perfusion, and collateral circulations. Detailed plans should be made based on preoperative evaluations, such as clipping method, aneurysm clip type, and whether it is necessary to expose the cervical carotid artery in advance or use intraoperative balloon-assisted occlusion, etc. (23). Giant aneurysms may cause ipsilateral cerebral ischemia and perfusion changes due to their blood steal and associated local hemodynamic changes, and preoperative CTP is necessary. As patients with abnormal cerebral perfusions have a lower tolerance to ischemia, temporary occlusion should be strictly timed. Indeed, neuroelectrophysiology and blood flow monitoring are more helpful. If delayed cerebral infarction occurs, medical treatment should be received promptly. The ACP, optic strut, and optic canal may reveal some variation. Some reports reveal that the ACP has three types of variations, namely, carotico-clinoidal foramen, interclinoid osseous bridge, and pneumatization, for which the variation percentages are 16.6%, 2.77%, and 27.7%, respectively. These variations increase the difficulty and risk of drilling the ACP using the Dolenc Approach. Therefore, a CT scan or skull three-dimensional reconstruction is needed before surgery to ensure the safe removal of the ACP (24–27).

Calcification increases difficulty or inability to perform aneurysm clipping. Therefore, it is a contraindication for clipping. Approximately 9% of ophthalmic aneurysms are calcified. Thereby, it is important to know whether the aneurysm neck is calcified before surgical treatment. Some scholars recommend that the aneurysm be clipped along the distal calcification when the aneurysm neck is calcified (28, 29). In our study, case 4 was a giant ophthalmic aneurysm that had a wide calcified aneurysm neck. Since we were unable to clip the neck, we placed aneurysm clips side by side on the aneurysm sac perpendicular to the parent artery along the calcified neck. Intraoperative ICG showed occlusion of the aneurysm and patency of the parent artery.

For those aneurysms that are considered complex prior to surgery, due to potential difficulty in performing the exposure or clipping, or whose proximal segment of the parent artery (clinoid segment and cavernous segment) has severe calcification, exposure of the cervical carotid artery in advance or intraoperative balloon-assisted occlusion is necessary to prevent uncontrolled bleeding during surgery (23). To reduce intraoperative risks, aneurysm trapping and ipsilateral extracranial-intracranial bypass is an alternative method (30). The application of multiple monitoring devices, such as DSA, ICG, neuroelectrophysiological monitoring, and ultrasonic, can be useful to monitor the mean blood flow velocity of MCA, ensure that the aneurysm is completely occluded, and prevent operation-related neurological impairment and ischemia during surgery (31). For case 3, preoperative CTA showed occlusion of the MCA, and superficial temporal artery-M3 segment bypass was performed before aneurysm clipping during surgery. The purpose of the bypass graft is to prevent exacerbation of cerebral ischemia during the surgical procedure. Postoperative CTA showed no aneurysm and no ipsilateral ICA, and the superficial temporal artery-middle cerebral artery anastomosis was unobstructed. The patient did not show any symptoms of ischemia after surgery.

Different aneurysm projections require the need for a different clipping technique and result in higher surgical risk. For example, giant aneurysms on the dorsal portion of the parent artery are usually proximal to the ACP and dura mater or may even adhere to the dura mater. Therefore, care should be taken during the removal of the ACP and when opening the dura mater to prevent aneurysm injury and rupture before the aneurysm neck is exposed. Some giant aneurysms originate from the ventral side of the parent artery, causing part of the neck and body to be in the blind field. Therefore, it is more difficult to expose the whole neck of these aneurysms and a fenestrated clip is needed to clip the aneurysm. Intraoperative DSA, ICG, or endoscopy is also essential to check if the aneurysm neck is completely occluded. Giant aneurysms can compress or adhere to the cavernous sinus when exposing the aneurysm neck or removing the aneurysm sac. Therefore, care should be taken to avoid excessive stretching to prevent cavernous sinus bleeding. Part of the aneurysm wall that adheres to the cavernous sinus can be reserved after clipping when the goal of decompression is achieved. If the aneurysm has a medial projection, it can compress the optic nerve, optic chiasm, hypothalamus, and pituitary. Care should be taken when removing the aneurysm to avoid injuring the pituitary stalk and hypothalamic perforating artery. In case 2, the patient complained of progressive visual impairment of the left eye, and the preoperative radiological examination showed a left giant ophthalmic aneurysm, with a narrow neck and long sac. During surgery, medial projection of the aneurysm was observed, which compressed and adhered the optic nerve and optic chiasm. The aneurysm neck was successfully clipped during surgery. Most of the thrombus was removed from space I and II, and part of the aneurysm was removed to reduce compression to the optic nerve and optic chiasm. The adhered aneurysm wall was not removed. The patient experienced diabetes insipidus and electrolyte imbalance after surgery but recovered after treatment. Indeed, injury of the pituitary stalk is a risk during surgery.

During clipping of giant C6–C7 segment aneurysms, the clip should be chosen according to the aneurysm location, projection, size, and neck width and its relationship with surrounding structures. Ventral side giant aneurysms are usually clipped by angled-fenestrated or T-shaped clip, as exposure is difficult in the surgical field due to limited surgical space and obstruction of the parent artery. A wide neck aneurysm in the medial wall of the parent artery could be clipped using an angled clip. For wide neck aneurysms, the clipping direction should be parallel to the parent artery or long axis of the aneurysm neck because vertical clipping can avulse the aneurysm neck or distort the parent artery. Medial narrow neck aneurysm can be clipped using a long straight clip or clipped perpendicularly to the parent artery. Single clipping is known to be the best way for small, narrow neck aneurysms. In our case study, three patients had giant ophthalmic aneurysms with narrow necks, of which, two aneurysms were a slender shape, and one was round in shape. A single aneurysm clip could be used to achieve complete occlusion of an aneurysm, which was the best way to clip aneurysms. Indeed, it was suitable for aneurysms with a small sac and narrow neck.

However, for those ophthalmic aneurysms and posterior communicating aneurysms with a giant sac, wide neck, special location, and close contact with surrounding structures, single clipping cannot achieve complete occlusion of the aneurysm. Instead, they usually need fenestrated clips or multiple clips. Clipping methods include the following: stacked clipping, tandem understacked clipping, tandem overstacked clipping, facing counter clipping, and crosswise counter clipping (32). The purpose is to achieve complete occlusion of the aneurysm, maintain the patency of the parent artery, and avoid injury of the vessel branches, such as the ophthalmic artery, superior pituitary artery, and posterior communicating artery. As the aneurysm is located in the ICA, the neck of the aneurysm is wide, bearing high tension from the blood flow, resulting in the aneurysm neck having a certain tension. After the aneurysm is occluded, sometimes an aneurysm clip will be added to enhance it to prevent incomplete occlusion, slippage, and recurrence. Multiple clipping and solid clipping are effective methods to achieve complete closure of aneurysms, reduce branch vessel damage, and prevent recurrence. In case 4, the parent artery wall of the aneurysm was part of the aneurysm neck, and the aneurysm was clipped multiple ways after the aneurysm was shaped.

Comparing giant or wide neck aneurysms with the common aneurysm, interventional treatment has a higher recurrence rate, and most times, it is difficult to perform interventional therapy again after the recurrence of interventional therapy; therefore, clipping is a better choice for surgical treatment (33–35). In this group, there were 13 cases of giant C6 aneurysm and 4 cases of giant C7 aneurysm. 3 cases of giant C6 aneurysm recurred after interventional therapy, 1 case of giant C7 segmental aneurysm recurred after interventional therapy, and 1 case of giant C7 segmental aneurysm recurred after interventional and surgical treatment. Recurrent giant aneurysms after interventional treatment also need multiple clipping or enhanced clipping. The removal of coiling material is still controversial since it can cause injury or avulsion of the parent artery wall and uncontrolled haemorrhage. In the following situations, interventional material can be removed. (1) In the event that interventional material results in difficult or incomplete aneurysm neck clipping. (2) The patient has obvious neurological dysfunction due to mass effect relevant to coiling material. After the removal of the interventional material and partial aneurysm, a residual part often has a jagged margin. Our experience is to clip the neck of the residual part of the aneurysm after suture of the break, as described in case 1. Clipping after suture has the advantages of being helpful in shaping the parent artery, achieving complete clipping, and preventing post-operative recurrence or the clip slipping off. This group included five cases of recurrent aneurysms, of which, four cases were clipped after removal of the interventional materials, and one case was clipped without removal of the interventional material due to satisfactory clipping and absence of space-occupying effects prior to surgery.

Giant aneurysms have a high incidence of thrombosis, and it can be diagnosed through pre-operative MRI examination (36). Research shows that for giant aneurysms with thrombus, clipping treatment is better than interventional treatment for complete occlusion of the aneurysm and to prevent recurrence (37). For aneurysms with thrombus and compression symptoms, the thrombus and partial aneurysm should be removed to relieve the pressure. Two patients in our study were revealed to have diabetes insipidus after surgery, which may be associated with iatrogenic injury of the pituitary stalk when removing the thrombus and a portion of the aneurysm.

Visual impairment is a common clinical characteristic of giant C6–C7 aneurysms, which is often the first symptom experienced by some patients during outpatient treatment. Visual impairment in the patient with a giant aneurysm is mostly caused by compression on the optic apparatus. Therefore, clipping and decompression can improve this symptom after surgery (38). In previous research that analyzed 2,458 cases of paraclinoid aneurysms, visual impairment incidence was 21%. Improvement by clipping is higher than embolization (58%−49%). However, clipping also causes a relatively higher incidence of visual impairment (11%−9%). For giant aneurysms, clipping does not reveal a statistically significant difference relative to interventional treatment in visual impairment (39). In a report that analysed 257 ophthalmic aneurysm cases, 38 cases with big or giant ophthalmic aneurysms underwent clipping surgeries. In 12 cases with pre-operative visual impairment, 9 cases improved, 2 cases experienced no postoperative change in symptoms, and visual impairment deteriorated after clipping in 1 case. There were also two patients with newly developed visual impairment (40). To date, there is no bulk data analysis on postoperative visual impairment of giant ophthalmic aneurysm. Among the 13 cases of giant ophthalmic aneurysms in our study, 1 had bilateral preoperative vision loss and 8 had ipsilateral pre-operative vision loss. Of the four patients with giant posterior communicating aneurysms, one had pre-operative oculomotor paralysis. Preoperative visual impairment is usually caused by aneurysm compression.

In addition, visual impairment is also a common postoperative complication of an aneurysm in this location. Among the 17 patients in our study, 5 had new visual impairment, 1 had ipsilateral vision loss, and 4 had ipsilateral oculomotor paralysis. New incidence and deterioration of visual impairment are related to surgical technique, as well as the size of the aneurysm (41). The following are some of the potential reasons for visual impairment caused by surgical technique. (1) Mechanical injury and heat conduction damage during drilling of the ACP. (2) Injury of the ophthalmic artery and oculomotor nerve when the aneurysm is exposed and clipped, or injury due to the perforation of arteries that irrigate the optic nerve. Therefore, nerve injury should be avoided when drilling the ACP. As giant aneurysms can compress the ophthalmic artery, optic nerve and oculomotor nerve, nerves and arteries should be protected when separating and exposing aneurysms. Retrograde suction for decompression of the aneurysm should be performed to reduce its size when necessary, making it easier to distinguish surrounding vessels or nerves and reduce injury of the optic nerve (42, 43). In addition, injuries and misclipping of aneurysms should be avoided as their neck is wide and they are proximal to the ophthalmic artery, superior pituitary artery, and posterior communicating artery. Both neuroelectrophysiological monitoring during surgery and ICG after clipping are necessary. Postoperative visual loss might be related to injury of the ophthalmic artery, superior pituitary artery, and optic nerve nutrient artery (44). Microvascular factors may cause delayed visual impairment after surgery. Also, injury or spasms of the superior ocular vein and optic nerve perforating vessels may cause optic nerve ischemia and delayed visual impairment (45).

Cerebral stroke is a common postoperative complication for giant C6–C7 aneurysm clipping. Related factors could include the following: (1) Prolonged temporary occlusion time; (2) Parent artery is narrow after aneurysm clipping; (3) Thrombus from aneurysm obstructs the distal artery; (4) Branch vessels are injured during surgery. Therefore, temporary occlusion should be strictly timed, and electrophysiology should be monitored. The application of intermittent occlusion should be performed during clipping when necessary. For those patients whose CTP shows cerebral hypoperfusion, more attention should be paid to temporary occlusion time and surgical monitoring. Indeed, the clipping technique is critical, as a giant aneurysm usually contains a thrombus, and clipping it in the wrong way could cause the thrombus to dislocate, contributing to a large thrombus embolization and massive infarction. Vessel injury during surgery is also an important reason for postoperative infarction. Therefore, branch vessels should be separated and protected when clipping the aneurysm, and care should be taken to avoid misclipping the aneurysm or causing injury to the vessel. ICG images after clipping can evaluate the flow of branch vessels. Monitoring the mean blood flow velocity of MCA has great significance. A giant aneurysm, whose neck is difficult to expose due to limited surgical space, may benefit from retrograde suction decompression after temporary occlusion, followed by clipping when its volume is reduced. Moreover, it is important to remove a thrombus from the aneurysm, protect the surrounding nerves and arteries, reduce surgery time, and prevent the occurrence of postoperative cerebrovascular events.

This study has some limitations. First, limited by the rare incidence of giant aneurysms, only 17 cases of giant aneurysms were included in this study, and the sample size was small. Secondly, this study is a descriptive study, which simply discusses the treatment of giant intracranial aneurysms by craniotomy and clipping, without comparing the effects of different treatment methods. Finally, this study is a single-centre study, and selection bias caused by geography cannot be avoided, and the results cannot represent the situation of other hospitals.

## 5. Conclusion

In this study, 17 cases of giant aneurysms were successfully clipped, and 9 cases had adverse postoperative complications. Giant ophthalmic aneurysms and posterior communicating artery aneurysms are complex intracranial aneurysms. The Dolenc approach is a safe, effective, and feasible way to clip these aneurysms, especially those with a wide neck, thrombus, compression, and recurrence. At the same time, neurosurgeons need to be familiar with the detailed anatomy of this region, as well as master microsurgical clipping of these aneurysms using the Dolenc approach.

## Data Availability

The raw data supporting the conclusions of this article will be made available by the authors, without undue reservation.
